# Global Economic Burden of Norovirus Gastroenteritis

**DOI:** 10.1371/journal.pone.0151219

**Published:** 2016-04-26

**Authors:** Sarah M. Bartsch, Benjamin A. Lopman, Sachiko Ozawa, Aron J. Hall, Bruce Y. Lee

**Affiliations:** 1 Public Health Computational and Operations Research, Johns Hopkins Bloomberg School of Public Health, Baltimore, MD, United States of America; 2 Division of Viral Diseases, National Center for Immunization and Respiratory Diseases, Centers for Disease Control and Prevention, Atlanta, GA, United States of America; 3 Department of International Health, Johns Hopkins Bloomberg School of Public Health, Baltimore, MD, United States of America; New York City Department of Health and Mental Hygiene, UNITED STATES

## Abstract

**Background:**

Despite accounting for approximately one fifth of all acute gastroenteritis illnesses, norovirus has received comparatively less attention than other infectious pathogens. With several candidate vaccines under development, characterizing the global economic burden of norovirus could help funders, policy makers, public health officials, and product developers determine how much attention and resources to allocate to advancing these technologies to prevent and control norovirus.

**Methods:**

We developed a computational simulation model to estimate the economic burden of norovirus in every country/area (233 total) stratified by WHO region and globally, from the health system and societal perspectives. We considered direct costs of illness (e.g., clinic visits and hospitalization) and productivity losses.

**Results:**

Globally, norovirus resulted in a total of $4.2 billion (95% UI: $3.2–5.7 billion) in direct health system costs and $60.3 billion (95% UI: $44.4–83.4 billion) in societal costs per year. Disease amongst children <5 years cost society $39.8 billion, compared to $20.4 billion for all other age groups combined. Costs per norovirus illness varied by both region and age and was highest among adults ≥55 years. Productivity losses represented 84–99% of total costs varying by region. While low and middle income countries and high income countries had similar disease incidence (10,148 vs. 9,935 illness per 100,000 persons), high income countries generated 62% of global health system costs. In sensitivity analysis, the probability of hospitalization had the largest impact on health system cost estimates ($2.8 billion globally, assuming no hospitalization costs), while the probability of missing productive days had the largest impact on societal cost estimates ($35.9 billion globally, with a 25% probability of missing productive days).

**Conclusions:**

The total economic burden is greatest in young children but the highest cost per illness is among older age groups in some regions. These large costs overwhelmingly are from productivity losses resulting from acute illness. Low, middle, and high income countries all have a considerable economic burden, suggesting that norovirus gastroenteritis is a truly global economic problem. Our findings can help identify which age group(s) and/or geographic regions may benefit the most from interventions.

## Introduction

Diarrheal disease remains the fourth most common cause of mortality and second most common cause of morbidity worldwide in children under the age of 5 years.[1] Despite accounting for approximately one-fifth of all acute gastroenteritis (AGE) worldwide across the age range[2], norovirus has received considerably less attention from the press and has fewer program initiatives than other high burden infectious pathogens. For example, programs that focus on rotavirus (e.g., Global Rotavirus Surveillance and Global Rotavirus Laboratory Networks, and ROTA Council) do not have equivalent counterparts for norovirus. Accordingly, norovirus may receive less funding and financial support. A systematic review of United Kingdom studies suggests that funding and attention from policy makers for norovirus is not proportional to its disease burden.[3]

There are several possible reasons for comparative lack of attention towards norovirus. First, disease burden has been challenging to estimate, in part because diagnostics of adequate sensitivity have not been widely available. Second, some associate norovirus primarily with outbreaks in cruise ships (which may be perceived to be of relatively minor health importance) and healthcare facilities[4]; however, of the estimated 20 million annual cases in the United States, only a small fraction (<1%) are associated with reported outbreaks.[5] Third, although norovirus is perceived to only cause self-limiting, mild gastroenteritis that rarely requires medical care, causes severe disease, or death, there are an estimated 70,000 norovirus-associated deaths among children <5 years annually worldwide.[6] Also, funding organizations and policy makers that focus on low- and middle-income countries (LMICs) may consider norovirus more of a priority for higher income countries, as those are the settings in which most norovirus data are generated.[2]

With a number of norovirus prevention and control measures currently under development (e.g., antiviral, disinfectants, and, most notably, vaccines[7]), understanding the global economic burden of norovirus is increasingly timely and critical. Decision makers such as funders, policy makers, public health officials, and product developers need more economic information to determine where norovirus should fall on their list of priorities and how much time, effort, and resources to invest. Moreover, without more information on the worldwide distribution of the economic burden, there may not be enough evidence on where and/or whom to target efforts and resources. For example, how does the economic burden of norovirus compare between high income countries and LMICs? To date, economic studies of norovirus quantified the impact of several outbreaks in hospital settings and community cases and determined the cost-effectiveness of a hypothetical norovirus vaccine, all in high income countries (including the United States, Switzerland, and Scotland).[8–12] To our knowledge, there has not been a systematic international assessment of the cost of norovirus. Thus, we developed a computational simulation model that can estimate the economic burden of norovirus in each country/area (i.e., territory/state) by WHO region and globally.

## Methods

### Model Structure

We constructed a computational simulation model in Microsoft Excel (Microsoft Corporation, Redmond, WA) with the Crystal Ball add-in (Oracle Corporation, Redwood Shore, CA) to estimate the economic burden of norovirus for any country, from the third party payer, health system, and societal perspectives. The model first determines the number of norovirus illnesses and norovirus-associated deaths in each of four age-groups (0 to 4 years old, 5 to 14 years old, 15 to 54 years old, and 55 years and older; referred to as young children, older children, adults, and older adults, respectively) in the specified country/area. Each norovirus case had probabilities of seeking medical care (i.e., outpatient or ambulatory care visits) and hospitalization. Additionally, each case had a probability of missing productive days (e.g., work or school days).

Third party payer or health system costs included all direct medical costs of illness (i.e., outpatient visits and hospitalization). Societal costs included direct and indirect (i.e., productivity losses due to absenteeism from work or school and mortality) costs. The cost per hospital bed day and duration of hospitalization were used to estimate hospitalization costs. Daily income served as a proxy for productivity losses associated with absenteeism and lost productive days due to norovirus illness and were accrued by all norovirus cases. A norovirus-specific premature death resulted in accruing the net present value of that person’s lifetime earnings, based on person’s age of death and his/her remaining years of life based on life expectancy. Costs are presented in 2013 US dollars, with past costs converted using country-specific Consumer Price Index (CPI) ratios, and future costs (future lifetime earnings) discounted at 3%, annually.

### Data Inputs and Sources

All inputs were age- and country-specific when available; Table 1 shows our model parameters, values, and sources at the regional and global level, while the S1 and S2 Tables provides country level inputs. We utilized data from the United Nations to determine the countries/areas included in our study and their 2010 population estimates.[13] The model calculates the number of norovirus cases and deaths in each country/area based on incidence data (median number of illnesses and deaths per 100,000 population; Table 1) from Foodborne Disease Epidemiology Reference Group (FERG).[14] FERG provides estimates of norovirus incidence and mortality by WHO region that account for the heterogeneity among countries within each region. Due to a lack of national incidence data for most countries our model utilized these region specific estimates of norovirus illness and deaths, modeled as distributions. The total number of cases was divided into age groups by applying the probability of a norovirus case being in a given age-group (Table 1). We calculated this distribution by normalizing the age-specific AGE incidence rate (i.e., dividing age-specific rates by total population rate) to determine the age-distribution of norovirus cases. Likewise, we determined the age distribution of norovirus deaths (Table 1). These probabilities were region- and WHO mortality stratum-specific and calculated by dividing the number of diarrheal deaths in each age-group by the total number of diarrheal deaths in the Global Burden of Disease 2010 data (i.e., we assumed that the age distribution of diarrhea deaths was representative of norovirus deaths in the absence of other data).[15] These calculated probabilities were applied to the total number of norovirus cases and deaths, in each location, thus determining the age distribution.

**Table 1:** Input values and sources.

**Regional Parameters**							
**Parameter**	**Africa**	**The Americas**	**Eastern Mediterranean**	**European**	**South-East Asia**	**Western Pacific**	**Source**
Total population	858,566,704	942,641,826	579,071,329	903,133,491	1,789,987,553	1,819,519,788	[13]
Number of norovirus cases per 100,000 persons^a^	11,268 (5,943–22,906)	14,906 (10,063–24,036)	18,203 (10,391–31,219)	6,408 (4,858–8,059)	5,343 (1,441–23,924)	8,320 (3,858–21,440)	[14]
Number of norovirus deaths per 100,000 persons^a^	7 (4–10)	0.8 (0.6–1.0)	3 (2–4)	0.2	7 (5–10)	0.3 (0.2–0.5)	[14]
Distribution of norovirus cases by age-group (%)							[47, 57]
0 to 4 year olds	73.1	67.7	54.4	61.9	65.3	64.5	
5 to 14 year olds	5.9	10.7	16.6	12.5	18.4	8.2	
15 to 54 years	9.8	13.1	16.6	8.2	8.1	13.0	
55 years and older	11.2	8.4	12.5	17.4	8.2	14.8	
Distribution of norovirus deaths by age-group (%)							[15]
In high mortality countries^b^							
0 to 4 year olds	63.3	57.9	72.3		27.5		
5 to 14 year olds	4.4	3.1	4.0		4.8		
15 to 54 years	16.3	13.4	10.5		19.3		
55 years and older	16.0	25.6	13.2		48.4		
In low mortality and developed countries^b^							
0 to 4 year olds		29.7	44.4	16.7	53.2	34.2	
5 to 14 year olds		1.7	2.4	0.7	3.7	1.8	
15 to 54 years		9.1	10.3	10.9	16.8	12.9	
55 years and older		59.6	42.9	71.7	26.3	51.1	
**Global Parameters**							
**Parameter**	**0 to 4 years old**	**5 to 14 years old**		**15 to 54 years old**		**55 years and older**	**Source**
Baseline ratio of seeking care^c^	1	0.794 (0.169)^d^		0.569 (0.184)^d^		0.569 (0.184)^d^	[16, 18–22, 25–27]
Probability of hospitalization per norovirus case (%)	0.428 (0.385–0.471)^e^	0.182 (0.164–0.201)^e^		0.486 (0.438–0.535)^e^		1.73 (1.56–1.91)^e^	[12]
Average duration of hospitalization (days)^f^	2.1–2.4	2.1		2.3–2.7		2.7–3.0	[58]

a) Values are median and 95% uncertainty interval

b) As defined by the WHO[59]

c) Compared to younger children (0–4 years old)

d) Values are mean (standard deviation)

e) Values are mean (range)

f) For ICD-9 codes 008.8 and 008.63

For young children in LMICs, the probability of seeking medical care for diarrhea (S2 Table) came from a study of Demographic Health Surveys (DHS) over 28 years (1985 to 2013) capturing nearly 1.4 million children across all datasets.[16] That study defined medical care seeking as a child taken to a medical facility, including all public and private medical facilities, however it did not include pharmacy or traditional healers which are also included in DHS surveys. The probability of seeking outpatient treatment for ages and countries not captured in the DHS datasets were derived from studies on the self-reported care seeking behavior for AGE (S2 Table).[17–30] For countries in which there were no data available, we created a distribution (S2 Table) from countries in the same WHO region with the same income classification for which data were available. These income classifications were defined according to the World Bank (low income: ≤$1,045; lower middle income: $1,046 to $4,125, upper middle income: $4,126 to $12,745, and high income: ≥$12,746).[31] For example, with no healthcare seeking behavior data available for high income countries in the European region, data from Italy, Poland, France, and Ireland (the 4 countries in which data were available) were used to generate distributions by age, that were then sampled for all other high income countries in the European region. In the absence of data, we assumed that older children (5 to 14 years old) and adults (15 years and older) were 79.4% and 56.9%, respectively, as likely to seek care as young children (0 to 4 years old; Table 1).[16, 18–22, 25–27] We derived these from studies in which data were reported for all age groups, where we calculated the proportion of older children and adults seeking care relative to younger children seeking care. We then created a distribution of weights by taking the mean and standard deviation across the calculated proportions. In the absence of hospitalization rates for many countries (especially LMICs), we derived the age-specific probability of hospitalization by dividing United States estimates of norovirus hospitalizations[32] by United States norovirus incidence[33, 34], as described in our previous study[12]. Hospitalization rates in other high income countries are similar to those seen in the United States[35], though data are lacking from lower income settings.

Gross national income (GNI) per capita (Atlas Method), used to estimate daily income (GNI/365 days, which inherently accounts for non-work days), came from the World Bank[36] and was supplemented with data from the UN[37] when not available (S1 Table). When not available from either source (3 countries and 17 areas), we utilized the average GNI from similar countries in the same region (defined by income classification when available or similar economies and industries). The cost of an outpatient visit and hospital bed day in US dollars (S1 Table) came from WHO Choice 2008.[38] For countries in which no estimates were available we calculated the average ratio of healthcare costs compared to the daily wage for that region and applied this to the daily wage for countries missing healthcare cost data in that region. In the absence of norovirus specific data, we assumed persons accrued missed productive days equal to the duration of illness, 2 days (range: 1 to 3).[39] Life expectancy data came from the WHO Global Health Observatory (WHO GHO)[40] and was country-specific when available. When not available, we used the life expectancy of corresponding WHO region for that country.

### Simulation Scenarios and Model Outcomes

In our baseline scenario we assumed that everyone with norovirus illness accrued lost productive days, used the healthcare seeking ratios found in Table 1, and used the probability of hospitalization from the United States. As there are no reliable data for hospitalization in LMICs and a majority of high income countries, an additional scenario calculated economic impacts excluding hospitalization. Additional scenarios evaluated the impact of various assumption on the proportion of productive days missed due to illness (25%, 50%, and 75%); in these scenario, productivity losses were accrued for the duration of an outpatient visit (half a day) if seeking care but not missing productive days. We varied other key parameters in sensitivity analysis to quantify the uncertainty resulting from unknowns regarding the influence of different national healthcare systems and access to care. Thus, additional one-way sensitivity analysis varied the following parameters: the probability of hospitalization (0% to distributions in Table 1), the ratio of care seeking behavior for older children (10% to 100%) and adults (10% to 70%), the probability of missing productivity days (25% to 100%), and daily income (+/- 20%).

For every simulation, the number of norovirus illnesses, deaths, and their associated costs were estimated for each country/area from each perspective and summed to give the total for all WHO regions, income strata, and worldwide. Simulation runs consisted of 10,000 probabilistic (i.e., Monte Carlo) trials, varying each parameter throughout their ranges to estimate uncertainty. We report the median and 95% uncertainty interval (95% UI), based on the 2.5^th^ and 97.5^th^ percentiles across the 10,000 Monte Carlo trials.

## Results

Globally, norovirus was estimated to cause a median number of 699 million illnesses [95% UI: 489–1,086 million] and 219,000 deaths (95% UI: 171,000–277,000) across all ages per year. These cases and deaths resulted in a median $4.2 billion (95% UI: $3.2–$5.7 billion) in direct health system costs (including hospitalization) and $56.2 billion (95% UI: $40.9–$78.3 billion) in productivity losses; thus costing society a total of $60.3 billion (95% UI: $44.4–$83.4 billion) annually. Approximately half of all productivity losses were due to mortality. When excluding hospitalization, norovirus resulted in a median $2.8 billion (95% UI: $2.1–$3.9 billion) in direct health system costs, costing society a total of $59.1 billion (95% UI: $43.4 to $81.8 billion).

Table 2 shows the breakdown of norovirus gastroenteritis illnesses, deaths, healthcare costs, productivity losses, and societal costs by age group. Disease amongst children <5 years accounted for approximately $39.8 billion (95% UI: $27.2–$58.1 billion) in total societal costs, compared to $20.4 billion (95% UI: $16.9–$25.4 billion) for all other age groups combined. This pattern was consistent across all regions. Globally, productivity losses accounted for 93% of the total economic burden. This proportion was fairly consistent for all regions and age groups (91% to 99%), except for European countries in which productivity losses represented 84% to 91% of the total burden.

**Table 2:** Number of norovirus illnesses and deaths [median (95% uncertainty interval)] and costs of norovirus disease [Median (95% uncertainty interval), $US in millions] for all countries and areas per year stratified by region with baseline assumptions.

Region	Norovirus Illnesses^a^	Norovirus Deaths^b^	Total Health System Costs^a^	Total Productivity Losses^a^	Total Societal Costs^a^
**All Ages**					
Africa	96.6 (51.0–195.5)	60.1 (34.3–85.9)	1289.9 (68.0–264.4)	3,855.6 (2,421.7–5,387.8)	3,992.2 (2,519.1–5,597.9)
The Americas	140.1 (94.7–223.9)	7.6 (5.6–9.4)	1,358.5 (907.6–2,192.4)	22,205.4 (14,368.4–35,916.2)	23,467.0 (15,294.8–37,742.4)
Eastern Mediterranean	105.4 (59.7–180.1)	17.3 (11.6–23.1)	362.2 (204.0–627.5)	4,893.8 (3,306.9–7,278.8)	5,253.9 (3,457.7–7,878.7)
European	57.9 (43.8–73.1)	1.8	1,162.9 (853.2–1,528.0)	7,968.2 (5,369.7–11,283.3)	9,135.8 (6,342.9–12,671.4)
South-East Asia	93.8 (27.9–435.2)	125.1 (89.3–178.6)	198.5 (58.9–920.8)	6,731.4 (4,644.2–10,528.5)	6,957.2 (4,753.1–11,293.1)
Western Pacific	152.4 (70.1–396.4)	5.5 (3.6–9.1)	758.5 (346.3–1,958.5)	8,933.7 (4,461.8–22,230.1)	9,695.0 (4,854.2–23,977.6)
Total Global Burden All Ages	698.8 (488.7–1,086.0)	218.8 (170.9–277.2)	4,182.1 (3,153.9–5,680.8)	56,181.1 (40,888.1–78,331.2)	60,268.9 (44,428.8–83,362.6)
**Young Children (Ages 0–4 years)**					
Africa	70.7 (37.3–143.0)	38.1 (21.7–54.4)	99.2 (52.0–201.1)	2,844.8 (1,787.3–4,040.2)	2,945.5 (1,860.9–4,201.0)
The Americas	94.9 (64.2–151.7)	2.4 (1.8–3.0)	830.3 (545.4–1,353.1)	15,161.0 (8,922.5–26,080.7)	15,992.1 (9,557.1–27,295.3)
Eastern Mediterranean	57.3 (32.5–98.0)	11.1 (7.5–14.8)	209.2 (118.2–358.9)	3,088.7 (2,048.4–4,699.8)	3,295.0 (2,192.3–5,025.2)
European	35.8 (27.1–45.2)	0.3	734.9 (513.0–1,010.4)	5,171.4 (3,121.5–7,787.4)	5,907.9 (3,749.0–8,678.0)
South-East Asia	61.3 (18.2–284.2)	40.3 (28.8–57.6)	139.6 (41.4–651.0)	3,456.5 (2,328.8–5,976.3)	3,605.8 (2,396.2–6,584.3)
Western Pacific	98.3 (45.2–255.6)	1.9 (1.2–3.1)	523.9 (239.8–1,357.0)	6,184.8 (2,804.5–16,587.9)	6,714.2 (3,087.7–17,837.8)
Total Global Burden Young Children	452.9 (315.3–705.5)	94.9 (72.9–118.3)	2,678.7 (2,000.8–3,685.5)	37,150.7 (24,830.6–54,835.0)	39,818.6 (27,214.6–58,143.6)
**Older Children (Ages 5–14 years)**					
Africa	5.7 (3.0–11.5)	2.7 (1.5–3.8)	6.0 (2.4–13.0)	205.1 (129.2–295.0)	211.4 (133.6–305.7)
The Americas	15.1 (10.2–24.1)	0.1 (0.1–0.2)	203.4 (135.5–327.5)	2,228.1 (1,237.9–3,967.3)	2,330.7 (1,318.2–4,111.3)
Eastern Mediterranean	17.5 (9.9–29.9)	0.6 (0.4–0.8)	45.7 (20.8–85.7)	572.4 (312.6–1,047.0)	618.7 (344.7–1,124.4)
European	7.2 (5.5–9.1)	0.0	100.1 (68.4–138.7)	1,007.8 (593.8–1,1536.5)	1,109.2 (678.6–1,655.4)
South-East Asia	17.2 (5.1–79.9)	5.7 (4.1–8.1)	31.4 (9.3–146.2)	504.2 (311.7–1,184.3)	535.2 (324.9–1,327.6)
Western Pacific	11.8 (5.4–30.8)	0.1 (0–0.2)	48.8 (19.5–133.7)	704.2 (299.5–1,954.5)	754.4 (324.8–2,08.4)
Total Global Burden Older Children	79.8 (54.7–142.0)	9.3 (7.2–12.0)	459.2 (335.3–639.8)	5,452.0 (3,431.9–8,241.4)	5,810.8 (3,754.9–8,680.3)
**Adults (Ages 15–54 years)**					
Africa	9.5 (5.0–19.2)	9.8 (5.6–14.0)	8.6 (3.4–20.0)	457.7 (280.4–634.2)	466.6 (288.4–645.0)
The Americas	18.4 (12.5–29.4)	0.7 (0.5–0.9)	122.5 (79.6–201.2)	1,748.6 (1,304.2–2,533.0)	1,869.5 (1,389.3–2,721.4)
Eastern Mediterranean	17.5 (9.9–29.9)	1.8 (1.2–2.4)	43.3 (20.0–86.1)	483.7 (340.4–676.9)	527.3 (368.4–751.5)
European	4.8 (3.6–6.0)	0.2	64.5 (48.5–81.8)	454.4 (374.5–540.7)	519.2 (423.0–621.3)
South-East Asia	7.6 (2.3–35.4)	23.6 (16.8–33.6)	10.8 (3.1–50.6)	1,092.6 (782.2–1,545.6)	1,107.1 (790.1–1,567.1)
Western Pacific	19.8 (9.1–51.5)	0.7 (0.5–1.2)	72.4 (28.3–207.1)	764.4 (429.1–1,666.6)	838.5 (463.6–1,866.0)
Total Global Burden Adults	83.4 (58.7–124.5)	37.0 (28.5–47.8)	339.9 (230.8–506.2)	5,114.7 (4,246.0–6,299.3)	5,454.9 (4,509.6–6,770.9)
**Older Adults (Ages 55 years and older)**					
Africa	10.8 (5.7–21.8)	9.6 (5.5–13.7)	15.8 (7.5–34.4)	342.7 (215.9–472.2)	360.2 (229.3–496.7)
The Americas	11.7 (7.9–18.7)	4.3 (3.2–5.3)	204.4 (135.4–327.2)	3,086.4 (2,404.8–3,817.6)	3,293.0 (2,567.3–4,107.0)
Eastern Mediterranean	13.1 (7.4–22.4)	3.8 (2.5–5.0)	62.9 (34.0–114.0)	745.1 (534.3–967.6)	809.1 (583.0–1,061.9)
European	10.1 (7.6–12.7)	1.3	261.8 (196.7–333.7)	1,338.7 (1,164.9–1,524.3)	1,601.0 (1,362.9–1,855.6)
South-East Asia	7.7 (2.3–35.6)	55.5 (39.7–79.2)	16.8 (5.0–78.2)	1,620.0 (1,160.9–2,291.7)	1,643.3 (1,176.6–2,325.3)
Western Pacific	22.5 (10.4–58.5)	2.8 (1.9–4.7)	111.1 (50.4–288.2)	1,253.1 (762.5–2,304.1)	1,361.9 (819.8–2,593.6)
Total Global Burden Older Adults	81.3 (57.4–124.6)	77.5 (60.5–101.7)	698.7 (536.2–928.1)	8,471.5 (7,328.7–9,872.0)	9,169.7 (7,916.4–10,712.4)

a) In millions

b) In thousands

**Fig 1:**
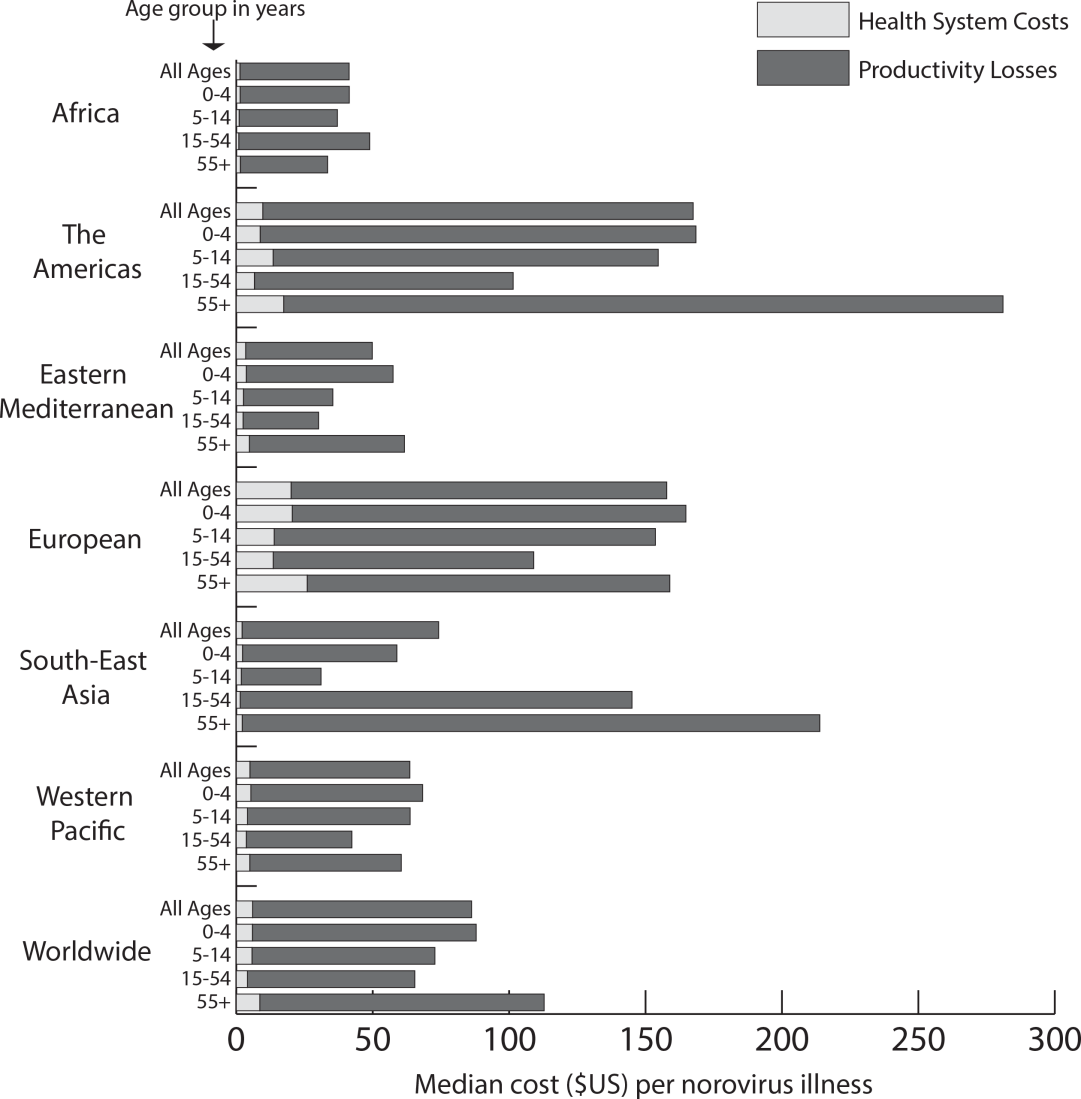
Median health system and productivity loss cost per norovirus illness by age group for each region and globally (total bar represents societal cost).

Fig 1 shows the median cost per norovirus illness by age group for each region and globally. Overall, norovirus cost $86 per illness globally. There are different age patterns in cost per illness in almost every region, largely due to variations in care seeking behavior across regions and the proportionally large number of deaths among older adults (compared to the other age groups). Health system costs for norovirus illness were highest among older adults in all regions except South-East Asia and the West Pacific, where health system costs were higher among young children. Despite health system costs being the highest among older adults in 4 regions, the societal costs were only highest among older adults in 2 of these regions (region of the Americas and Eastern Mediterranean). Older adults in the European region had the highest health system cost per illness at $25.98, but older adults in the region of the Americas had the highest societal illness costs ($281.02).

Globally, LMICs have a greater cumulative norovirus disease burden (82% of total global illness and 97% of global deaths) compared to high income countries, but norovirus-associated costs were consistently higher in high income countries (Table 3). However, the number of illnesses per 100,000 persons were similar for LMICs and high income countries (10,148 vs. 9,935), thus the large cumulative disease burden in LMICs reflects that ~82% of the global population lives in LMICs. Sixty-two percent of global health system costs were generated by high income countries. Norovirus cost society $45 and $274 per illness in LMICs and high income countries, respectively, with productivity losses driving much of the cost as health system costs were $3 and $20 per illness in LMICs and high income countries, respectively. Thus, productivity losses represented 93% of total costs in high income countries and 94% in LMIC.

**Table 3:** Number of norovirus illnesses and deaths [median (95% uncertainty interval)] and costs of norovirus disease [Median (95% uncertainty interval), $US in millions] for low and middle income countries and high income countries per year stratified by region with baseline assumptions.

Region	Norovirus Illnesses^a^	Norovirus Deaths^b^	Total Health System Costs^a^	Total Productivity Losses^a^	Total Societal Costs^a^
Low and Middle Income Countries	570.3 (380.8–943.9)	212.5 (164.8–270.8)	1,580.2 (1,070.1–2,531.4)	23,846.0 (18,085.4–32,914.2)	25,438.5 (19,350.0–35,173.9)
High Income Countries	126.5 (97.0–168.5)	6.3 (5.4–7.3)	2,574.4 (1,970.7–3,369.0)	32,209.9 (22,166.8–46,556.6)	34,660.2 (24,310.0–49,573.2)
Total Global Burden	698.8 (488.7–1,086.0)	218.8 (170.9–277.2)	4,182.1 (3,153.9–5,680.8)	56,181.1 (40,888.1–78,331.2)	60,268.9 (44,428.8–83,362.6)

Total population among LMIC = 5,620,076,521; High income countries = 1,272,916,170

a) In millions

b) In thousands

**Fig 2:**
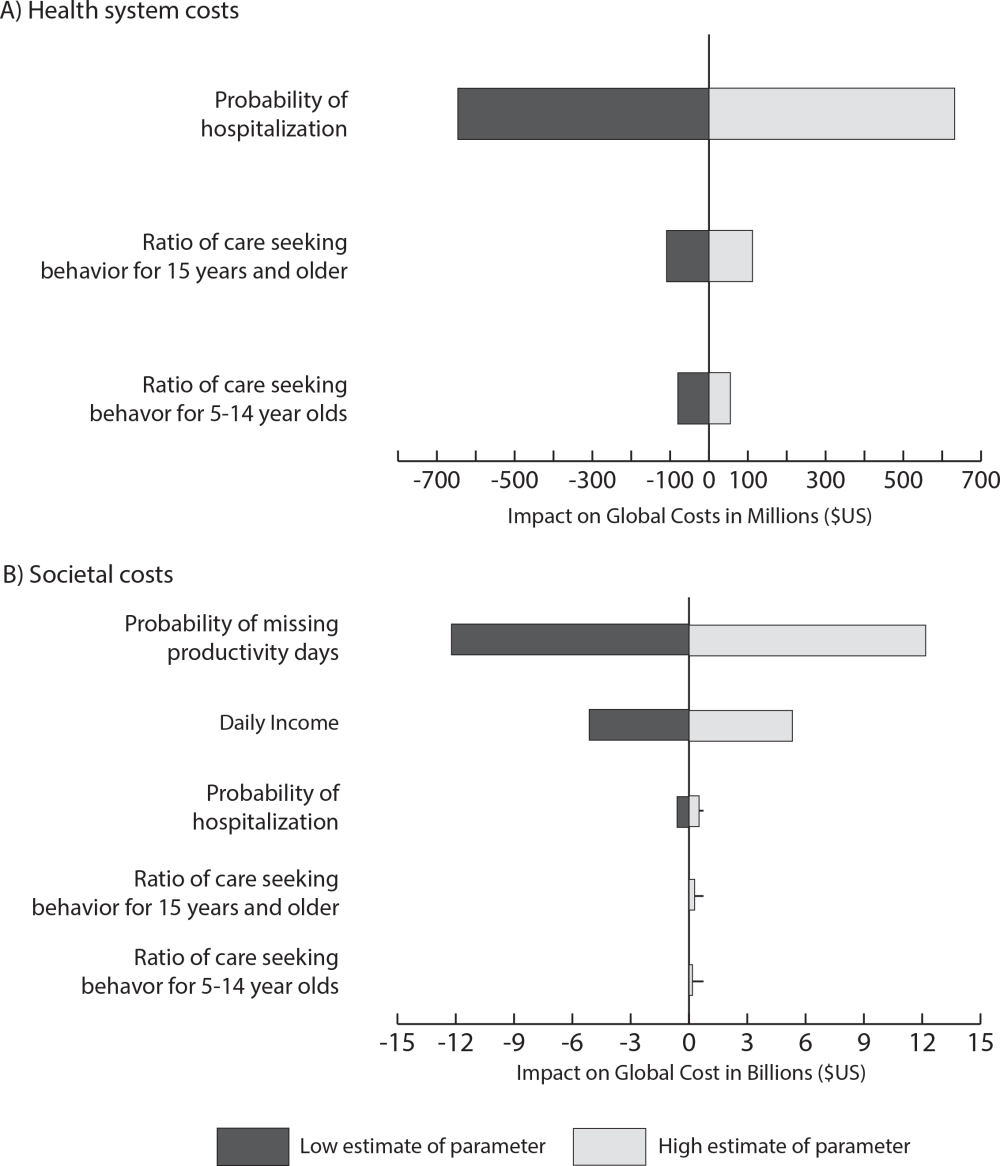
Impact of key parameters on the total health system (a) and societal (b) costs while holding all other values constant. Zero indicates point at which all variable list on the y-axis are held at median values; the x-axis shows the magnitude of the impact on total cost for each parameter.

Fig 2 shows the magnitude of impact that key parameters had on estimates of the total global healthcare costs and societal costs. In this tornado diagram, the x-axis shows the deviation in costs from a base case in which all parameters on the y-axis are held at their median values (e.g., half of the distribution value for the probability of hospitalization, 63% probability of missing productivity days). Using median values for these parameters, norovirus generated a total of $3.4 billion (95% CI: $2.6–$4.6 billion) in health system costs and $47.2 billion (95% UI: $36.6–$62.7 billion) in societal costs globally. The width of the bar shows the range of the impact each parameter had when varied from its minimum to maximum value. Globally, total healthcare costs were most affected by the probability of hospitalization (Fig 2A), while societal costs were most affected by the probability of missing productive days (Fig 2B). The patterns illustrated in Fig 2 were similar for most regions; except for Africa, where care seeking in adults and older children had a larger impact on societal costs than the probability of hospitalization, and South-East Asia, where care seeking in older children had a larger impact on societal costs (after missed productivity days and income).

Further exploring the impact of missing productive days, global societal costs were $51.8 billion (95% UI: $39.5 –$69.8 billion), $43.9 billion (95% UI: $34.6 –$57.2 billion) and $35.9 billion (95% UI: $29.7 –$44.2 billion) when the probability of missing productive days was 75%, 50%, and 25%, respectively. While societal costs decreased (as a result of lower productivity losses), productivity losses still represented a substantial portion of the total burden as pre-mature mortality resulted in $17.8 billion globally. Even when only 25% of illnesses missed productivity days, productivity losses represented 86% and 91% of the total burden in high income and LMICs, respectively, as they are largely driven by pre-mature mortality.

## Discussion

Worldwide, we estimate that the approximately 699 million norovirus illness and 219,000 deaths result in $4.2 billion (95% CI: $3.2–$5.7 billion) in health system costs and $60.3 billion (95% CI: $44.4–$83.4 billion) in societal costs annually. Productivity losses represent the largest portion of this economic burden in all regions–of which, half of losses were due to norovirus deaths. As most persons with AGE do not seek medical care and therefore do not incur healthcare costs, overlooking productivity losses would severely underestimate the true cost of norovirus illness. Total cost was most sensitive to hospitalization rates, probability of missing productive days, and care seeking rates. Lacking better data on how norovirus affects productivity, our baseline scenario assumed that everyone would lose productivity time each day ill (1 to 3 days). However, it may be unlikely that everyone with norovirus have a productivity decrement, thus additional scenarios evaluated the impact of this parameter. These scenarios also serve as a proxy for uncertainty in the labor force, especially in LMICs where productivity losses may be less of an issue if only one parent works. Total societal costs were still considerable, $35.9 billion (95% UI: $29.7 –$44.2 billion), when only 25% of illnesses missed productive days, as half of all productivity losses are due to norovirus mortality.

Our results suggest that the economic burden of norovirus gastroenteritis may exceed that of other diseases that have received more attention. For example, the annual societal cost of rotavirus in LMICs is an estimated $423 million ($262 million to $590 million) and direct medical treatment costs results in an estimated $325 million ($202 to $453 million) in the absence of vaccination (2007 values)[41]. Globally, rotavirus is estimated to cost $2 billion annually (2007 values).[42] Although the general methodologies of these studies are similar (identifying the number of cases and associated unit costs), caution should be taken when making comparisons as there is variation in specific costs included. For example, diagnostic test and medication costs were included and only productivity losses for caregivers were considered in the LMIC cost estimates. Besides the differences in methodology, norovirus may also be more costly as it is practically ubiquitous, affecting persons of all ages, in all locations worldwide, and with the potential for multiple episodes throughout life. This contrasts with other pathogens, such as rotavirus, which predominately affect young children. While the global economic burden of rotavirus may be lower than norovirus, rotavirus is more clearly established as a cause of death in young children, which is likely part of the reason hence it has been identified for decades as an important vaccine target.

Our results also demonstrate the considerable economic burden of norovirus in both high income countries and LMICs, highlighting that norovirus is a truly global problem. When determining global and LMIC priorities, focusing on mortality and major chronic outcomes can overlook diseases such as norovirus AGE, for which severe outcomes are uncommon. Such self-limiting but high incidence diseases still result in productivity losses that cumulatively can lead to considerable burden to society. Tracking productivity losses can be challenging, but ultimately are important to consider. LMICs with fewer material resources and human capital may be particularly susceptible to productivity losses.[43] As our study shows, productivity losses represent 94% of the total burden in LMIC when 100% of illnesses accrue productivity losses and 91% when only 25% miss productive days. These productivity losses likely represent a large portion of a household’s income in LMICs. For example, productivity losses accounted for 73% of spending for rotavirus in Malaysia and households with the lowest income are more likely to experience catastrophic payments than those with the highest income[44]; in Bolivia, indirect costs equate to 3 to 4 days of income[45] and in Kenya direct and indirect costs represent a large part of a households monthly income[46].

These results can inform funding agencies and public heath bodies regarding where to best allocate limited resources and gauge investments and potential returns for interventions and control measures. Without additional investment and attention, some efforts may not be realized. This is especially important with vaccines for norovirus, which are in the development pipeline.[7] Different vaccine candidates are at various stages of clinical development with a bivalent intramuscular formulation expected to soon progress to Phase III clinical trials. Our analysis clearly shows that the total economic burden is greatest in young children but that the highest cost per illness is among older age groups in some regions. These findings can be used to help identify which age group and/or geographic regions may benefit the most from a norovirus vaccine. However, it should be noted that the data underlying the illness burden estimates in adults and children over 5 years of age are limited, especially in LMICs, and are based on very few studies[2, 14, 47].

### Limitations

All models, by definition, are simplifications of reality[48] and therefore cannot account for every possible event or outcome. Our model attempted to be conservative about certain costs of norovirus. Our intention to estimate the economic burden based on established health burden estimates and it should be noted that some studies exclude vomiting-only norovirus episodes, which may represent 13–27% of cases in the community[49, 50], and therefore underestimate the true cost burden of all norovirus illnesses. We did not include the costs of any treatment administered outside the formal health system (e.g., traditional healers, oral rehydration) as their use may vary by region and symptoms and there is limited data on cost and usage. We also excluded losses due to long term growth impairment[51] and chronic disorders that may be a consequence of norovirus infection (e.g., post-infectious irritable bowel syndrome, constipation, dyspepsia, and gastroesophageal reflux disease)[52]. Additionally, we did not model the potential impact of malnutrition, which may be a risk factor for an increased duration of diarrhea[53], as its impact is not well quantified outside of a few small studies in specific locations (e.g., rural and slum areas) that show a longer duration[54–56]. Even though our model utilized distributions for norovirus illness and death that accounted for heterogeneity across countries in each WHO region, our results may overestimate the number of each in some countries and underestimate in others. For example, the rate of norovirus in the United States and Canada most likely falls at the lower end of the reported distribution for the region of the Americas. Additionally, the age distribution of AGE deaths may not be representative for norovirus, especially if a large portion were due to rotavirus, which disproportionally causes disease and deaths in children <5 years. We assumed all norovirus episodes had the same likelihood of seeking care, and we made broad generalizations about the probability of hospitalization and although varied in sensitivity analysis, this parameter has a large impact on costs. Our estimates can be refined as more and better data become available. More reliable data on care seeking behavior, hospitalization rates for AGE, and missed productivity, especially for LMICs and older children and adults would be particularly useful. It should be noted that data inputs for our model derived from different hierarchical levels (e.g., country, region, globe, and ages) that may introduce unknown bias.

### Conclusions

Our results present an economic argument for greater consideration of norovirus. The large cost of norovirus overwhelmingly are from productivity losses resulting from acute illness. Thus, these results point to the importance of taking a broader view of economic impact that includes productivity losses. Productivity losses tend to go unrecognized, but make up 94% of the global economic burden of norovirus. Focusing only on medically-attended outcomes substantially underestimates the total economic impact of norovirus illness. Additionally, low, middle and high income countries all have a considerable economic burden, suggesting that norovirus gastroenteritis is a truly global economic problem.

Disclaimer: The findings and conclusions in this report are those of the authors and do not necessarily represent the official position of the Centers for Disease Control and Prevention, or the US Department of Health and Human Services.

## Supporting Information

S1 Table Country/Area Level Input Parameters, Values, and Sources. (DOCX)

S2 Table Country and Regional Level Care Seeking Behavior Input Values and Sources. (DOCX)
