# Supplementary material for: Global Economic Burden of Norovirus Gastroenteritis
Source: PLoS One. 2016 Apr 26;11(4):e0151219. doi: 10.1371/journal.pone.0151219 (PMC4846012; doi:10.1371/journal.pone.0151219)
Supplement: S1 Table — (DOCX) [file pone.0151219.s001.docx]

**S1 Table. Country/Area Level Input Parameters, Values, and Sources**

| **Country/Area** | **Total Population[1]** | **Mortality Stratum** | **GNI per Capita (2013)[2]** | **Cost of Outpatient Visit[3]** | **Cost of Hospital Bed Day[3]** | **Lifetime Productivity Losses[2, 4]** | | | |
| --- | --- | --- | --- | --- | --- | --- | --- | --- | --- |
|  |  |  |  |  |  | **0-4 years** | **5-14 years** | **15-54 years** | **55 years and older** |
| **Africa** |  |  |  |  |  |  |  |  |  |
| Algeria | 37,062,820 | High | 5,330 | 8.98 | 59.63 | 153,719 | 151,936 | 117,963 | 84,627 |
| Angola | 19,549,124 | High | 5,170 | 10.43 | 60.82 | 144,581 | 144,581 | 112,529 | 79,224 |
| Benin | 9,509,798 | High | 790 | 1.59 | 6.05 | 22,910 | 22,654 | 17,765 | 12,106 |
| Botswana | 1,969,341 | High | 7,770 | 12.25 | 96.79 | 228,849 | 225,333 | 174,726 | 127,545 |
| Burkina Faso | 15,540,284 | High | 670 | 1.21 | 4.51 | 19,323 | 19,099 | 14,828 | 9,885 |
| Burundi | 9,232,753 | High | 260 | 0.47 | 1.15 | 7,412 | 7,319 | 5,754 | 3,984 |
| Cameroon | 20,624,343 | High | 1,290 | 2.29 | 9.92 | 36,773 | 36,315 | 28,078 | 20,482 |
| Cape Verde | 487,601 | High | 3,620 | 5.87 | 30.89 | 111,141 | 109,491 | 89,419 | 63,146 |
| Central African Republic | 4,349,921 | High | 320 | 1.03 | 3.10 | 8,888 | 8,760 | 6,720 | 4,904 |
| Chad | 11,720,781 | High | 1,020 | 1.72 | 6.33 | 28,330 | 28,330 | 21,420 | 14,449 |
| Comoros | 683,081 | High | 840 | 1.68 | 5.87 | 24,617 | 24,226 | 18,889 | 12,872 |
| Congo, Rep. | 4,111,715 | High | 2,590 | 5.70 | 29.87 | 74,697 | 73,830 | 58,242 | 41,123 |
| Cote d’Ivoire | 18,976,588 | High | 1,450 | 2.13 | 8.41 | 40,550 | 39,988 | 30,451 | 22,220 |
| Democratic Republic of the Congo | 62,191,161 | High | 430 | 0.49 | 1.13 | 12,025 | 11,943 | 9,359 | 6,344 |
| Equatorial Guinea | 696,167 | High | 14,320 | 42.09 | 444.75 | 408,203 | 400,465 | 311,686 | 219,437 |
| Eritrea | 5,741,159 | High | 490 | 0.80 | 2.26 | 14,432 | 14,210 | 10,845 | 7,229 |
| Ethiopia | 87,095,281 | High | 470 | 0.96 | 3.08 | 13,910 | 13,774 | 11,041 | 7,715 |
| Gabon | 1,556,222 | High | 10,650 | 14.16 | 113.77 | 313,673 | 308,854 | 243,163 | 174,820 |
| The Gambia | 1,680,640 | High | 500 | 1.09 | 4.04 | 14,578 | 14,338 | 11,416 | 7,939 |
| Ghana | 24,262,901 | High | 1,770 | 2.07 | 7.85 | 51,872 | 51,331 | 41,006 | 28,103 |
| Guinea | 10,876,033 | High | 460 | 1.14 | 3.86 | 13,267 | 13,113 | 10,344 | 7,049 |
| Guinea-Bissau | 1,586,624 | High | 590 | 0.62 | 1.70 | 16,609 | 16,500 | 12,619 | 8,705 |
| Kenya | 40,909,194 | High | 1,160 | 1.84 | 7.10 | 33,821 | 33,455 | 26,485 | 19,041 |
| Lesotho | 2,008,921 | High | 1,500 | 1.76 | 6.80 | 41,062 | 40,095 | 28,991 | 22,986 |
| Liberia | 3,957,990 | High | 410 | 0.64 | 1.56 | 12,016 | 11,825 | 9,220 | 6,283 |
| Madagascar | 21,079,532 | High | 440 | 1.25 | 4.23 | 13,022 | 12,828 | 10,046 | 6,986 |
| Malawi | 15,013,694 | High | 270 | 0.97 | 2.98 | 7,787 | 7,649 | 5,877 | 4,137 |
| Mali | 13,985,961 | High | 670 | 1.39 | 4.87 | 19,323 | 19,213 | 14,828 | 10,267 |
| Mauritania | 3,609,420 | High | 1,060 | 2.14 | 9.12 | 31,371 | 30,905 | 24,202 | 16,830 |
| Mauritius | 1,230,659 | High | 9,290 | 11.19 | 85.11 | 284,208 | 279,847 | 226,791 | 166,621 |
| Mozambique | 23,967,265 | High | 610 | 1.06 | 3.35 | 17,059 | 16,822 | 12,566 | 9,348 |
| Namibia | 2,178,967 | High | 5,870 | 7.84 | 48.07 | 175,320 | 172,888 | 137,901 | 99,420 |
| Niger | 15,893,746 | High | 400 | 1.13 | 3.34 | 11,600 | 11,536 | 8,995 | 6,130 |
| Nigeria | 159,707,780 | High | 2,710 | 2.55 | 10.96 | 76,777 | 75,786 | 58,985 | 41,527 |
| Rwanda | 10,836,732 | High | 630 | 1.00 | 3.42 | 18,645 | 18,463 | 14,800 | 10,341 |
| Sao Tome and Principe | 178,228 | High | 1,470 | 2.42 | 10.00 | 43,905 | 43,296 | 34,534 | 24,897 |
| Senegal | 12,950,564 | High | 1,050 | 2.53 | 10.25 | 31,075 | 30,613 | 23,974 | 16,090 |
| Seychelles | 91,208 | High | 13,210 | 21.90 | 200.81 | 402,650 | 397,930 | 322,488 | 230,430 |
| Sierra Leone | 5,751,976 | High | 660 | 0.76 | 2.36 | 17,642 | 17,642 | 13,324 | 8,539 |
| South Africa | 51,452,352 | High | 7,190 | 10.27 | 73.39 | 207,362 | 203,700 | 153,785 | 110,178 |
| South Sudan* | 9,940,929 | High | 950 | 1.77 | 7.76 | 27,081 | 26,743 | 20,678 | 14,558 |
| Swaziland | 1,193,148 | High | 2,990 | 4.83 | 27.08 | 83,046 | 81,227 | 59,095 | 47,474 |
| Tanzania | 44,973,330 | High | 630 | 1.34 | 4.84 | 18,463 | 18,169 | 14,384 | 10,341 |
| Togo | 6,306,014 | High | 530 | 1.32 | 4.67 | 15,285 | 15,108 | 11,730 | 8,415 |
| Uganda | 33,987,213 | High | 550 | 1.34 | 4.95 | 15,772 | 15,483 | 11,971 | 8,733 |
| Zambia | 13,216,985 | High | 1,810 | 2.99 | 11.01 | 51,903 | 51,279 | 40,059 | 28,738 |
| Zimbabwe | 13,076,978 | High | 860 | 1.6 | 6.8 | 24,803 | 24,515 | 19,033 | 14,117 |
| Mayotte*^ | 204,353 | High | 9,184 | 17.11 | 74.98 | 264,870 | 261,798 | 203,259 | 145,819 |
| Reunion*^ | 844,579 | High | 9,184 | 17.11 | 74.98 | 264,870 | 261,798 | 203,259 | 145,819 |
| Western Sahara*^ | 514,648 | High | 2,497 | 4.65 | 20.39 | 72,014 | 71,179 | 55,263 | 39,646 |
| **The Americas** | | | | | | | | | |
| Antigua and Barbuda | 87,233 | Low | 13,050 | 18.98 | 170.60 | 400,659 | 394,711 | 322,353 | 240,292 |
| Argentina | 40,374,224 | Low | 14,504 | 13.38 | 106.77 | 446,833 | 440,415 | 362,338 | 267,064 |
| The Bahamas | 360,498 | Low | 21,570 | 26.89 | 254.45 | 662,238 | 652,407 | 532,808 | 397,172 |
| Barbados | 280,396 | Low | 14,317 | 21.06 | 193.99 | 443,970 | 437,998 | 365,351 | 276,705 |
| Belize | 308,595 | Low | 4,510 | 7.01 | 44.71 | 138,465 | 136,410 | 111,403 | 80,889 |
| Bolivia | 10,156,601 | High | 2,550 | 3.15 | 16.92 | 76,161 | 75,105 | 59,906 | 43,189 |
| Brazil | 195,210,154 | Low | 11,690 | 2.39 | 20.36 | 358,904 | 353,576 | 288,759 | 215,250 |
| Canada | 34,126,240 | Developed | 52,200 | 54.72 | 607.67 | 1,633,443 | 1,613,517 | 1,358,490 | 1,053,837 |
| Chile | 17,150,760 | Low | 15,230 | 14.65 | 117.55 | 473,756 | 467,589 | 392,560 | 301,007 |
| Colombia | 46,444,798 | Low | 7,590 | 16.09 | 58.45 | 235,366 | 232,200 | 195,636 | 150,010 |
| Costa Rica | 4,669,685 | Low | 9,550 | 11.28 | 83.19 | 297,070 | 293,202 | 246,156 | 184,573 |
| Cuba | 11,281,768 | Developed | 6,884 | 10.04 | 53.36 | 212,786 | 209,829 | 173,850 | 129,948 |
| Dominica | 71,167 | Low | 6,930 | 8.20 | 57.62 | 212,009 | 208,755 | 169,178 | 127,603 |
| Dominican Republic | 10,016,797 | Low | 5,770 | 7.68 | 50.97 | 176,521 | 173,812 | 142,527 | 106,244 |
| Ecuador | 15,001,072 | High | 5,760 | 6.82 | 45.39 | 177,452 | 174,903 | 145,465 | 108,731 |
| El Salvador | 6218,195 | Low | 3,720 | 5.53 | 34.62 | 112,958 | 111,105 | 90,814 | 68,497 |
| Grenada | 104,677 | Low | 7,490 | 9.80 | 66.82 | 228,300 | 224,678 | 180,620 | 130,653 |
| Guatemala | 14,341,576 | High | 3,340 | 5.02 | 27.98 | 101,419 | 100,190 | 82,503 | 61,500 |
| Guyana | 786,126 | Low | 3,750 | 2.72 | 13.10 | 109,899 | 107,533 | 81,622 | 53,123 |
| Haiti | 9,896,400 | High | 810 | 1.44 | 5.01 | 23,857 | 23,490 | 18,494 | 12,861 |
| Honduras | 7,621,204 | Low | 2,180 | 3.70 | 19.16 | 66,692 | 65,936 | 54,461 | 41,152 |
| Jamaica | 2,741,485 | Low | 5,220 | 11.24 | 73.14 | 159,695 | 157,244 | 128,941 | 96,117 |
| Mexico | 117,886,404 | Low | 9,940 | 15.99 | 128.13 | 305,176 | 300,646 | 248,320 | 183,027 |
| Nicaragua | 5,822,209 | High | 1,790 | 1.95 | 8.98 | 54,761 | 53,921 | 44,215 | 32,105 |
| Panama | 3,678,128 | Low | 10,700 | 11.43 | 87.90 | 330,740 | 326,143 | 273,050 | 206,799 |
| Paraguay | 6,459,721 | Low | 4,010 | 4.64 | 25.77 | 123,114 | 121,287 | 100,177 | 73,837 |
| Peru | 29,262,830 | High | 6,270 | 7.33 | 49.36 | 193,808 | 191,114 | 158,344 | 118,358 |
| St. Kitts and Nevis | 4,849 | Low | 13,890 | 15.41 | 125.15 | 423,376 | 418,414 | 339,088 | 242,292 |
| St. Lucia | 177,397 | Low | 7,060 | 8.66 | 61.31 | 216,755 | 213,537 | 176,372 | 129,997 |
| St. Vincent and the Grenadines | 109,316 | Low | 6,460 | 7.90 | 54.75 | 197,630 | 194,597 | 159,571 | 115,864 |
| Suriname | 524,960 | Low | 9,370 | 9.76 | 62.79 | 288,667 | 285,604 | 236,633 | 181,094 |
| Trinidad and Tobago | 1,328,095 | Low | 15,760 | 32.31 | 315.66 | 474,745 | 468,593 | 370,241 | 258,701 |
| United States | 312,247,116 | Developed | 53,470 | 55.92 | 659.15 | 1,658,104 | 1,635,801 | 1,364,486 | 1,033,416 |
| Uruguay | 3,371,982 | Low | 15,180 | 16.84 | 129.01 | 467,659 | 460,942 | 379,225 | 279,512 |
| Venezuela, RB | 29,043,283 | Low | 12,550 | 41.41 | 317.45 | 386,635 | 381,082 | 316,941 | 242,554 |
| Anguilla*† | 14,612 | Low/Developed | 19,732 | 26.49 | 186.51 | 607,896 | 601,445 | 498,317 | 372,478 |
| Aruba* | 101,597 | Low/Developed | 23,649 | 31.75 | 223.53 | 728,569 | 720,837 | 597,238 | 446,418 |
| Bermuda* | 65,124 | Low/Developed | 108,457 | 145.60 | 1025.14 | 3,341,300 | 3,305,842 | 2,739,003 | 2,047,326 |
| British Virgin Islands*^† | 29,685 | Low/Developed | 30,319 | 40.70 | 286.58 | 934,058 | 924,146 | 765,686 | 572,328 |
| Caribbean Netherlands*^† | 19,196 | Low/Developed | 16,443 | 22.07 | 155.42 | 506,569 | 501,194 | 415,256 | 310,392 |
| Cayman Islands* | 55,509 | Low/Developed | 53,850 | 72.29 | 508.99 | 1,658,989 | 1,641,384 | 1,359,943 | 1,016,518 |
| Curacao* | 147,560 | Low/Developed | 19,759 | 26.53 | 186.76 | 608,727 | 602,268 | 498,999 | 372,988 |
| French Guiana*^ | 231,169 | Low/Developed | 11,203 | 15.04 | 105.89 | 345,138 | 341,475 | 282,924 | 211,477 |
| Greenland* | 56,905 | Low/Developed | 41,434 | 55.62 | 391.64 | 1,276,482 | 1,262,936 | 1,046,386 | 782,143 |
| Guadeloupe*^ | 428,335 | Low/Developed | 16,443 | 22.07 | 155.42 | 506,569 | 501,194 | 415,256 | 310,392 |
| Martinique*^ | 400,810 | Low/Developed | 16,443 | 22.07 | 155.42 | 506,569 | 501,194 | 415,256 | 310,392 |
| Montserrat*† | 4,737 | Low/Developed | 10,899 | 14.63 | 103.02 | 335,772 | 332,209 | 275,246 | 205,739 |
| Puerto Rico* | 3,709,671 | Low/Developed | 19,210 | 25.79 | 181.57 | 591,814 | 585,534 | 485,135 | 362,624 |
| Saint Pierre and Miquelon*^ | 5,515 | Low/Developed | 16,443 | 22.07 | 155.42 | 506,569 | 501,194 | 415,256 | 310,392 |
| Sint Maarten (Dutch part)* | 37,850 | Low/Developed | 22,572 | 30.30 | 213.35 | 695,389 | 688,010 | 570,039 | 426,088 |
| St. Martin (French part)*^ | 30,235 | Low/Developed | 16,443 | 22.07 | 155.42 | 506,569 | 501,194 | 415,256 | 310,392 |
| Turks and Caicos Islands* | 30,993 | Low/Developed | 23,348 | 31.34 | 220.69 | 719,296 | 711,663 | 589,637 | 440,737 |
| Virgin Islands*^ | 106,382 | Low/Developed | 16,443 | 22.07 | 155.42 | 506,569 | 501,194 | 415,256 | 310,392 |
| **Eastern Mediterranean** | | | | | | | | | |
| Afghanistan | 28,397,812 | High | 690 | 1.36 | 4.18 | 20,221 | 20,010 | 15,516 | 10,573 |
| Bahrain | 1,251,513 | Low | 21,477 | 37.32 | 399.25 | 663,860 | 654,633 | 536,536 | 385,202 |
| Djibouti | 834,036 | High | 1,781 | 2.22 | 9.61 | 52,195 | 51,365 | 40,050 | 28,278 |
| Egypt, Arab Republic | 78,075,705 | High | 3,140 | 4.46 | 25.93 | 94,973 | 93,362 | 74,758 | 51,543 |
| Iran, Islamic Republic | 74,462,314 | Low | 5,780 | 14.01 | 102.88 | 176,827 | 174,113 | 142,774 | 103,667 |
| Iraq | 30,962,380 | High | 6,720 | 6.45 | 31.88 | 203,253 | 199,806 | 159,991 | 110,309 |
| Jordan | 6,454,554 | Low | 4,950 | 6.05 | 34.84 | 151,435 | 149,111 | 120,841 | 86,346 |
| Kuwait | 2,991,580 | Low | 55,809 | 66.25 | 783.91 | 1,730,637 | 1,707,358 | 1,409,416 | 1,027,619 |
| Lebanon | 4,341,092 | Low | 9,870 | 10.73 | 80.60 | 307,024 | 303,027 | 254,404 | 190,758 |
| Libya | 6,040,612 | Low | 11,956 | 22.58 | 189.17 | 367,071 | 361,622 | 295,329 | 208,556 |
| Morocco | 31,642,360 | High | 3,020 | 4.61 | 24.92 | 91,343 | 90,198 | 71,901 | 49,573 |
| Oman | 2,802,768 | Low | 20,662 | 40.21 | 401.57 | 636,547 | 627,403 | 516,176 | 370,584 |
| Pakistan | 173,149,306 | High | 1,360 | 1.39 | 6.17 | 40,796 | 40,249 | 31,950 | 22,324 |
| Qatar | 1,749,713 | Low | 86,790 | 109.03 | 1541.75 | 2,699,758 | 2,664,612 | 2,214,769 | 1,638,321 |
| Saudi Arabia | 27,258,387 | Low | 26,260 | 23.98 | 224.08 | 806,230 | 794,261 | 648,658 | 458,069 |
| Somalia* | 9,636,173 | High | 128 | 0.22 | 1.51 | 3,626 | 3,626 | 2,833 | 1,961 |
| Sudan | 35,652,002 | High | 1,550 | 5.42 | 23.19 | 45,652 | 44,951 | 35,909 | 25,443 |
| Syrian Arab Republic | 21,532,647 | Low | 1,573 | 5.58 | 29.38 | 48,294 | 47,577 | 38,855 | 28,213 |
| Tunisia | 10,631,830 | Low | 4,200 | 6.98 | 46.27 | 129,392 | 127,533 | 104,924 | 75,329 |
| United Arab Emirates | 8,441,537 | Low | 43,085 | 69.69 | 741.53 | 1,327,345 | 1,308,280 | 1,076,345 | 772,753 |
| Yemen | 22,763,008 | High | 1,330 | 3.00 | 13.63 | 39,361 | 38,777 | 30,367 | 20,381 |
| **European** |  |  |  |  |  |  |  |  |  |
| Albania | 3,150,143 | Developed | 4,710 | 6.48 | 43.41 | 144,092 | 141,881 | 114,982 | 82,159 |
| Andorra | 77,907 | Developed | 41,014 | 112.83 | 237.68 | 1,283,410 | 1,267,755 | 1,077,303 | 828,009 |
| Armenia | 2,963,496 | Developed | 3,800 | 6.87 | 41.17 | 114,935 | 112,986 | 89,271 | 60,334 |
| Austria | 8,401,924 | Developed | 50,430 | 61.38 | 683.23 | 1,573,455 | 1,553,627 | 1,312,426 | 996,704 |
| Azerbaijan | 9,094,718 | Developed | 7,350 | 8.74 | 60.37 | 224,033 | 220,479 | 177,244 | 124,486 |
| Belarus | 9,491,070 | Developed | 6,730 | 19.48 | 148.03 | 203,556 | 200,103 | 158,104 | 110,473 |
| Belgium | 10,941,288 | Developed | 46,290 | 58.49 | 635.86 | 1,439,933 | 1,421,188 | 1,193,146 | 894,648 |
| Bosnia and Herzegovina | 3,845,929 | Developed | 4,780 | 7.18 | 49.39 | 147,260 | 145,145 | 119,413 | 85,732 |
| Bulgaria | 7,389,175 | Developed | 7,360 | 9.94 | 78.37 | 225,164 | 221,708 | 179,675 | 128,385 |
| Croatia | 4,338,027 | Developed | 13,430 | 21.82 | 193.66 | 415,125 | 409,355 | 339,165 | 247,289 |
| Cyprus | 1,103,685 | Developed | 25,210 | 39.94 | 408.85 | 788,871 | 779,248 | 656,083 | 498,253 |
| Czech Republic | 10,553,701 | Developed | 18,950 | 27.17 | 262.71 | 585,750 | 577,609 | 478,568 | 348,929 |
| Denmark | 5,550,959 | Developed | 61,680 | 76.79 | 846.59 | 12,025 | 11,943 | 9,359 | 6,344 |
| Estonia | 1,298,533 | Developed | 17,690 | 24.25 | 221.74 | 543,115 | 535,052 | 441,930 | 325,729 |
| Finland | 5,367,693 | Developed | 48,820 | 62.71 | 685.98 | 1,518,633 | 1,498,863 | 1,258,357 | 964,884 |
| France | 63,230,866 | Developed | 43,460 | 54.81 | 583.39 | 1,359,950 | 1,343,361 | 1,131,034 | 877,390 |
| Georgia | 4,388,674 | Developed | 3,570 | 4.89 | 27.59 | 109,217 | 107,541 | 87,152 | 62,274 |
| Germany | 83,017,404 | Developed | 47,270 | 54.54 | 593.74 | 1,474,860 | 1,456,275 | 1,218,405 | 934,249 |
| Greece | 11,109,999 | Developed | 22,690 | 39.43 | 399.89 | 707,945 | 699,024 | 590,501 | 448,448 |
| Hungary | 10,014,633 | Developed | 13,260 | 22.96 | 207.34 | 405,662 | 399,437 | 323,708 | 231,302 |
| Iceland | 318,042 | Developed | 46,400 | 78.73 | 870.43 | 1,451,949 | 1,434,237 | 1,218,775 | 936,744 |
| Ireland | 4,467,561 | Developed | 43,110 | 66.38 | 761.42 | 1,345,065 | 1,328,116 | 1,121,925 | 852,031 |
| Israel | 7,420,368 | Developed | 33,930 | 36.78 | 364.08 | 1,061,738 | 1,048,786 | 891,229 | 684,994 |
| Italy | 60,508,978 | Developed | 35,860 | 48.65 | 508.11 | 1,125,308 | 1,112,018 | 941,924 | 723,958 |
| Kazakhstan | 15,921,127 | Developed | 11,550 | 15.02 | 111.57 | 344,964 | 338,490 | 259,727 | 176,990 |
| Kyrgyz Republic | 5,334,223 | Developed | 1,210 | 2.06 | 8.83 | 36,449 | 35,810 | 28,032 | 19,212 |
| Latvia | 2,090,519 | Developed | 15,280 | 20.00 | 173.08 | 465,745 | 458,356 | 368,474 | 266,538 |
| Lithuania | 3,068,457 | Developed | 14,900 | 19.75 | 175.78 | 454,162 | 446,957 | 359,310 | 259,910 |
| Luxembourg | 507,885 | Developed | 69,900 | 131.40 | 1903.01 | 2,187,311 | 2,160,629 | 1,819,127 | 1,381,511 |
| Macedonia, FYR* | 424,738 | Developed | 4,870 | 7.16 | 63.38 | 149,518 | 147,298 | 118,888 | 84,950 |
| Malta | 2,102,216 | Developed | 20,980 | 22.22 | 279.11 | 654,592 | 646,343 | 540,769 | 405,481 |
| Moldova | 3,573,024 | Developed | 2,470 | 3.27 | 15.64 | 74,405 | 73,100 | 57,223 | 39,217 |
| Monaco | 36,845 | Developed | 173,377 | 213.21 | 3748.27 | 5,425,314 | 5,359,133 | 4,554,043 | 3,500,214 |
| Montenegro | 620,078 | Developed | 7,250 | 10.70 | 84.56 | 222,588 | 219,284 | 179,085 | 126,466 |
| Netherlands | 16,615,243 | Developed | 51,060 | 60.85 | 695.50 | 1,593,111 | 1,573,036 | 1,328,822 | 1,009,155 |
| Norway | 4,891,251 | Developed | 102,610 | 114.57 | 1468.98 | 3,201,510 | 3,161,168 | 2,670,395 | 2,027,995 |
| Poland | 38,198,754 | Developed | 13,240 | 18.45 | 159.02 | 407,893 | 402,034 | 327,046 | 243,790 |
| Portugal | 10,589,792 | Developed | 21,260 | 29.27 | 281.19 | 663,328 | 654,970 | 547,986 | 420,185 |
| Romania | 21,861,476 | Developed | 9,060 | 16.70 | 136.62 | 277,172 | 272,918 | 221,176 | 158,039 |
| Russian Federation | 143,617,913 | Developed | 13,850 | 18.65 | 167.30 | 413,658 | 407,923 | 320,866 | 219,903 |
| San Marino | 40,680 | Developed | 48,987 | 105.41 | 1281.86 | 1,537,241 | 1,519,086 | 1,286,727 | 988,972 |
| Serbia | 7,871,429 | Developed | 6,050 | 10.13 | 68.58 | 185,087 | 182,247 | 145,894 | 102,468 |
| Slovak Republic | 5,433,437 | Developed | 17,810 | 23.26 | 217.10 | 546,800 | 538,682 | 439,931 | 319,432 |
| Slovenia | 2,054,232 | Developed | 23,210 | 39.29 | 395.92 | 721,989 | 712,589 | 598,248 | 448,580 |
| Spain* | 46,182,038 | Developed | 29,920 | 44.02 | 389.37 | 936,257 | 924,836 | 785,900 | 604,039 |
| Sweden | 9,382,297 | Developed | 61,760 | 61.92 | 687.45 | 1,926,959 | 1,909,019 | 1,607,286 | 1,220,631 |
| Switzerland | 7,830,534 | Developed | 90,760 | 73.84 | 857.58 | 2,848,102 | 2,814,467 | 2,383,966 | 1,832,304 |
| Tajikistan | 7,627,326 | Developed | 990 | 1.65 | 6.85 | 29,944 | 29,436 | 23,258 | 15,719 |
| Turkey | 72,137,546 | Developed | 10,970 | 18.47 | 146.11 | 336,799 | 331,799 | 274,051 | 201,992 |
| Turkmenistan | 5,041,995 | Developed | 6,880 | 6.84 | 42.54 | 202,636 | 199,522 | 152,267 | 105,428 |
| Ukraine | 46,050,220 | Developed | 3,960 | 7.22 | 46.33 | 119,289 | 117,196 | 91,742 | 65,003 |
| United Kingdom | 62,066,350 | Developed | 41,680 | 56.43 | 615.00 | 1,300,448 | 1,284,061 | 1,074,321 | 823,768 |
| Uzbekistan | 27,769,270 | Developed | 1,880 | 2.09 | 9.72 | 56,632 | 55,639 | 44,166 | 29,850 |
| Channel Islands* | 159,518 | Developed | 78,139 | 114.96 | 1016.88 | 2,407,275 | 2,372,698 | 1,952,061 | 1,438,785 |
| Faeroe Islands*^ | 49,581 | Developed | 16,534 | 24.33 | 215.17 | 509,373 | 502,056 | 413,051 | 304,443 |
| Gibraltar*^† | 29,180 | Developed | 16,534 | 24.33 | 215.17 | 509,373 | 502,056 | 413,051 | 304,443 |
| Holy See* | 872 | Developed | 35,860 | 48.65 | 508.11 | 1,125,308 | 1,112,018 | 941,924 | 723,958 |
| Isle of Man* | 83,992 | Developed | 54,643 | 80.39 | 711.12 | 1,683,420 | 1,659,239 | 1,365,086 | 1,006,150 |
| Kosovo* | 1,775,680 | Developed | 3,940 | 5.80 | 51.27 | 121,382 | 119,638 | 98,429 | 72,548 |
| Liechtenstein* | 36,120 | Developed | 119,918 | 176.43 | 1560.58 | 3,694,386 | 3,641,321 | 2,995,780 | 2,208,068 |
| State of Palestine* | 4,012,880 | Developed | 3,134 | 4.61 | 40.78 | 96,551 | 95,164 | 78,293 | 57,707 |
| **South & South East Asia** |  |  |  |  |  |  |  |  |  |
| Bangladesh | 151,125,475 | High | 1,010 | 1.18 | 4.35 | 30,669 | 30,166 | 24,356 | 17,106 |
| Bhutan | 716,939 | High | 2,330 | 3.70 | 20.59 | 69,893 | 68,956 | 55,473 | 40,644 |
| India | 1,205,624,648 | High | 1,570 | 2.68 | 12.75 | 46,891 | 46,241 | 36,373 | 24,928 |
| Indonesia | 240,676,485 | Low | 3,580 | 4.33 | 22.77 | 108,281 | 106,444 | 85,233 | 58,766 |
| Korea, Dem Rep | 24,500,520 | High | 622 | 1.30 | 5.03 | 18,737 | 18,408 | 14,612 | 9,876 |
| Maldives | 325,694 | High | 5,600 | 7.91 | 46.50 | 173,098 | 170,692 | 141,424 | 103,114 |
| Myanmar | 51,931,231 | High | 1,183 | 1.08 | 3.80 | 35,174 | 34,670 | 27,407 | 18,783 |
| Nepal | 26,846,016 | High | 730 | 0.83 | 2.85 | 21,898 | 21,604 | 17,149 | 11,591 |
| Sri Lanka | 20,758,779 | Low | 3,170 | 52.35 | 547.83 | 96,979 | 95,491 | 78,303 | 56,856 |
| Thailand | 66,402,316 | Low | 5,340 | 6.24 | 41.86 | 163,366 | 161,514 | 131,905 | 98,326 |
| Timor-Leste | 1,079,450 | High | 3,847 | 1.34 | 4.33 | 115,399 | 113,305 | 89,124 | 61,081 |
| **Western Pacific** |  |  |  |  |  |  |  |  |  |
| Australia | 22,404,488 | Developed | 65,390 | 62.05 | 690.49 | 2,046,184 | 2,027,743 | 1,717,580 | 1,320,123 |
| Brunei Darussalam | 400,569 | Developed | 38,750 | 42.11 | 504.12 | 1,193,794 | 1,176,647 | 968,049 | 695,002 |
| Cambodia | 14,364,931 | Low | 950 | 1.46 | 6.21 | 28,957 | 28,617 | 23,733 | 18,361 |
| China | 1,359,821,465 | Low | 6,560 | 5.44 | 32.88 | 201,404 | 198,414 | 162,041 | 114,430 |
| Hong Kong SAR* | 7,049,514 | Low/Developed | 38,420 | 44.42 | 281.04 | 1,179,564 | 1,162,053 | 949,026 | 670,183 |
| Macao SAR* | 534,626 | Low/Developed | 81,848 | 94.63 | 598.72 | 2,512,883 | 2,475,578 | 2,021,757 | 1,427,724 |
| Cook Islands† | 13,067 | Low | 16,002 | 18.50 | 117.06 | 491,291 | 483,997 | 395,271 | 294,647 |
| Fiji | 860,559 | Low | 4,370 | 7.62 | 42.02 | 131,639 | 129,330 | 101,241 | 69,385 |
| Japan | 127,352,833 | Developed | 46,330 | 44.72 | 472.42 | 1,453,863 | 1,436,693 | 1,227,822 | 954,418 |
| Kiribati | 97,743 | Low | 2,620 | 2.83 | 12.60 | 78,592 | 77,539 | 61,550 | 43,007 |
| Korea, Rep. | 48,453,931 | Low | 25,920 | 25.42 | 251.61 | 808,724 | 798,533 | 674,560 | 512,286 |
| Lao PDR | 6,395,713 | Low | 1,450 | 1.73 | 7.30 | 43,307 | 42,913 | 34,064 | 23,022 |
| Malaysia | 28,275,835 | Low | 10,430 | 11.56 | 92.06 | 317,913 | 312,870 | 251,517 | 176,652 |
| Marshall Islands | 52,428 | Low | 4,310 | 4.83 | 19.55 | 130,874 | 129,288 | 103,935 | 72,998 |
| Micronesia, Fed. Sts. | 103,619 | Low | 3,280 | 4.59 | 22.12 | 98,805 | 97,072 | 77,055 | 53,841 |
| Mongolia | 2,712,738 | Low | 3,770 | 4.59 | 23.10 | 112,599 | 110,485 | 86,078 | 57,771 |
| Nauru | 10,025 | Low | 15,737 | 9.83 | 45.71 | 489,527 | 484,819 | 405,628 | 304,149 |
| New Zealand | 4,368,136 | Developed | 40,318 | 45.78 | 451.27 | 1,261,631 | 1,246,241 | 1,059,021 | 813,958 |
| Niue^† | 1,534 | Low | 4,900 | 12.95 | 110.49 | 67,362 | 66,294 | 53,951 | 37,431 |
| Palau | 20,470 | Low | 10,970 | 14.57 | 100.41 | 333,105 | 329,068 | 264,539 | 185,798 |
| Papua New Guinea | 6,858,945 | Low | 2,010 | 2.38 | 10.29 | 58,906 | 57,969 | 44,485 | 29,655 |
| Philippines | 93,444,322 | Low | 3,270 | 3.31 | 16.72 | 98,091 | 96,311 | 75,757 | 51,919 |
| Samoa | 186,029 | Low | 3,970 | 5.18 | 28.34 | 121,008 | 119,089 | 95,736 | 67,240 |
| Singapore | 5,078,969 | Developed | 54,040 | 46.40 | 573.65 | 1,695,807 | 1,675,780 | 1,419,453 | 1,090,984 |
| Solomon Islands | 526,447 | Low | 1,600 | 2.18 | 10.14 | 47,995 | 47,125 | 37,068 | 25,404 |
| Tonga | 104,098 | Low | 4,490 | 5.45 | 29.65 | 135,805 | 133,501 | 105,481 | 73,703 |
| Tuvalu | 9,827 | Low | 5,840 | 5.28 | 24.24 | 174,424 | 172,005 | 133,340 | 89,491 |
| Vanuatu | 236,299 | Low | 3,130 | 4.55 | 24.54 | 95,043 | 93,484 | 74,520 | 53,013 |
| Vietnam | 89,047,397 | Low | 1,740 | 2.34 | 10.95 | 53,605 | 52,835 | 43,469 | 32,846 |
| American Samoa*^ | 55,636 | Low/Developed | 6,539 | 7.56 | 47.83 | 201,451 | 198,557 | 163,357 | 120,404 |
| French Polynesia* | 268,065 | Low/Developed | 23,162 | 31.09 | 218.93 | 713,566 | 703,316 | 578,631 | 426,485 |
| Guam*^ | 159,440 | Low/Developed | 46,230 | 53.45 | 338.18 | 123,538 | 121,764 | 100,177 | 73,837 |
| New Caledonia* | 246,379 | Low/Developed | 37,862 | 43.78 | 276.97 | 1,166,437 | 1,149,683 | 945,865 | 697,159 |
| Northern Mariana Islands*^ | 53,860 | Low/Developed | 46,230 | 53.45 | 338.18 | 123,538 | 121,764 | 100,177 | 73,837 |
| Pitcairn*^† | 62 | Low/Developed | 46,230 | 53.45 | 338.18 | 123,538 | 121,764 | 100,177 | 73,837 |
| Tokelau*^† | 1,724 | Low/Developed | 3,633 | 4.20 | 26.58 | 111,924 | 110,316 | 90,759 | 66,895 |
| Wallis and Futuna Islands*^† | 20,065 | Low/Developed | 3,633 | 4.20 | 26.58 | 111,924 | 110,316 | 90,759 | 66,895 |

*Country not available in WHO Choice. Cost of outpatient visit and hospital bed day calculated by applying the average ratio of healthcare costs compared to the daily wage for that region and to the daily wage. Calculated outpatient visit ratios: Africa = 0.68; The Americas = 0.49; Eastern Mediterranean = 0.628; European = 0.537; Western Pacific = 0.422. Calculated hospital bed day ratios: Africa = 2.98; The Americas = 3.45; Eastern Mediterranean = 4.317; European = 4.75; Western Pacific = 2.67.

^Estimated GNI per capita (average GNI from similar countries in the same region, defined by income classification when available or similar economies and industries).

†Total population estimates from the UN Population Prospects supplemented with population data from the World Bank and CIA World Fact Book.

**References**

1. United Nations, Department of Economic and Social Affairs, Population Division. World Population Prospects: The 2012 Revision, DVD Edition 2013.

2. The World Bank. GNI per capita, Atlas method (current US$): The World Bank; 2015 [cited 2015 November]. Available from: <http://data.worldbank.org/indicator/NY.GNP.PCAP.CD>.

3. World Health Organization. Choosing Interventions that are Cost Effective (WHO-CHOICE): WHO-CHOICE unit cost estimates for service delivery Geneva, Switzerland: World Health Organization; 2011 [cited 2013 May 15]. Available from: <http://www.who.int/choice/country/country_specific/en/index.html>.

4. World Health Organization. Global Health Observatory: Country Statistics Geneva, Switzerland: World Health Organization; 2013 [updated 2014; cited 2015 May 12]. Available from: <http://www.who.int/gho/countries/en/#M>.
