# Supplementary material for: Global Economic Burden of Norovirus Gastroenteritis
Source: PLoS One. 2016 Apr 26;11(4):e0151219. doi: 10.1371/journal.pone.0151219 (PMC4846012; doi:10.1371/journal.pone.0151219)
Supplement: S2 Table — (DOCX) [file pone.0151219.s002.docx]

**S2 Table. Country and Regional Level Care Seeking Behavior Input Values and Sources^•^**

|  | **0-4 years old** | **5-14 years old** | **15 years and older** | **Source** |
| --- | --- | --- | --- | --- |
| **Country Specific Data** | | | | |
| Albania | 60.98 |  |  | [1] |
| Armenia | 33.27 |  |  | [1] |
| Australia | 17.0 (2.8 – 24.7)^ | 49.1 (24.2 – 62.6)^ | 21.0 (2.2 – 49.7)^ | [2] |
| Azerbaijan | 33.77 |  |  | [1] |
| Bangladesh | 27.12 |  |  | [1] |
| Benin | 22.57 |  |  | [1] |
| Bolivia | 38.79 |  |  | [1] |
| Brazil | 28.85 |  |  | [1] |
| Burkina Faso | 27.11 |  |  | [1] |
| Burundi | 57.41 |  |  | [1] |
| Cambodia | 39.63 |  |  | [1] |
| Cameroon | 22.92 |  |  | [1] |
| Canada | 33.3 (14.4 – 52.3)^ | 44.6 (10.0 – 64.3)^ | 19.6 (4.6 – 53.1)† | [2] |
| Central African Republic | 30.0 |  |  | [1] |
| Chad | 18.12 |  |  | [1] |
| China | 75.07 |  |  | [3, 4] |
| Colombia | 35.39 |  |  | [1] |
| Comoros | 29.71 |  |  | [1] |
| Republic of the Congo | 33.13 |  |  | [1] |
| Democratic Republic of the Congo | 32.6 |  |  | [1] |
| Cote d’Ivoire | 26.39 |  |  | [1] |
| Dominican Republic | 41.74 |  |  | [1] |
| Egypt | 48.24 |  |  | [1] |
| Ethiopia | 20.82 |  |  | [1] |
| France | 40.3 | 42.2 | 28.1 | [5] |
| Gabon | 34.85 |  |  | [1] |
| Gambia | 39.5 |  |  | [6] |
| Ghana | 29.83 |  |  | [1] |
| Guatemala | 26.05 |  |  | [1] |
| Guinea | 39.09 |  |  | [1] |
| Guyana | 58.66 |  |  | [1] |
| Haiti | 26.98 |  |  | [1] |
| Honduras | 49.34 |  |  | [1] |
| India | 62.61 | 50 | 29.17 | [1, 7] |
| Indonesia | 55.59 | 60.0 | 49.0 | [1, 8] |
| Iran | 70.0 |  |  | [9] |
| Ireland | 26.4 (16.5 – 39.7)^ | 15.8 (9.3 – 25.6)^ | 23.8 (4.6 – 54.9)† | [2] |
| Italy | 72 | 52.4 | 31.8 | [10] |
| Jordan | 52.24 |  |  | [1] |
| Kazakhstan | 25.74 |  |  | [1] |
| Kenya | 40.64 |  |  | [1] |
| Kyrgyzstan | 46.23 |  |  | [1] |
| Lesotho | 40.02 |  |  | [1] |
| Liberia | 50.1 |  |  | [1] |
| Madagascar | 35.59 |  |  | [1] |
| Malawi | 44.85 |  |  | [1] |
| Malaysia | 48.61 | 34.87 | 31.03 | [11] |
| Maldives | 84.57 |  |  | [1] |
| Mali | 15.82 |  |  | [1] |
| Moldova | 40.17 |  |  | [1] |
| Morocco | 16.54 |  |  | [1] |
| Mozambique | 48.31 |  |  | [1] |
| Namibia | 61.26 |  |  | [1] |
| Nepal | 23.16 |  |  | [1] |
| New Zealand | 39.5 |  |  | [12] |
| Nicaragua | 44.28 |  |  | [1] |
| Niger | 24.17 |  |  | [1] |
| Nigeria | 26.17 |  |  | [1] |
| Pakistan | 59.37 |  |  | [1] |
| Paraguay | 37.25 |  |  | [1] |
| Peru | 30.81 |  |  | [1] |
| Philippines | 35.78 |  |  | [1] |
| Poland | 80 | 52.6 | 27.3 | [13] |
| Rwanda | 22.05 |  |  | [1] |
| Sao Tome Principe | 52.21 |  |  | [1] |
| Senegal | 27.78 |  |  | [1] |
| Sierra Leone | 47.75 |  |  | [1] |
| South Africa | 61.42 |  |  | [1] |
| Swaziland | 56.85 |  |  | [1] |
| Tanzania | 54.92 |  |  | [1] |
| Timor-Leste | 72.43 |  |  | [1] |
| Togo | 18.52 |  |  | [1] |
| Turkey | 31.02 |  |  | [1] |
| Uganda | 62.51 |  |  | [1] |
| United Arab Emirates | 22.0 |  |  | [14] |
| United States | 16.8 (10.0 – 23.5)† | 16.8 (11.1 – 22.6)† | 6.4 (5.4 – 10.3)† | [15] |
| Vietnam | 54.77 |  |  | [1] |
| Yemen | 29.42 |  |  | [1] |
| Zambia | 49.38 |  |  | [1] |
| Zimbabwe | 32.82 |  |  | [1] |
| **Regional Estimates*** | | | | |
| Africa |  |  |  |  |
| Low Income | 35 (16 – 63)† |  |  |  |
| Lower Middle Income | 37.4 (22.9 – 56.8)† |  |  |  |
| Upper Middle Income | 52.3 (34.8 – 61.4)† |  |  |  |
| The Americas |  |  |  |  |
| Lower Middle Income | 42.4 (26.1 – 58.7)† |  |  |  |
| Upper Middle Income | 34.2 (28.9 – 41.7)† |  |  |  |
| High Income | 25.0‡ |  |  |  |
| Eastern Mediterranean |  |  |  |  |
| Lower Middle Income | 38 (17 – 59)† |  |  |  |
| Upper Middle Income | 52.24 |  |  |  |
| High Income | 22.0 |  |  |  |
| European |  |  |  |  |
| Lower Middle Income | 39.9 (33.3 – 46.2)† |  |  |  |
| Upper Middle Income | 38.0 (25.7 – 61.0)† |  |  |  |
| High Income | 55.0 (27.0 – 80.0)† | 41 (16 – 53)† | 29 (27 – 32)† |  |
| South-East Asia |  |  |  |  |
| Low Income | 25.14 |  |  |  |
| Lower Middle Income | 64 (56 – 72)† |  |  |  |
| Upper Middle Income | 84.57 |  |  |  |
| Western Pacific |  |  |  |  |
| Low Income | 39.63 |  |  |  |
| Lower Middle Income | 45.28 |  |  |  |
| High Income | 27.7‡ |  |  |  |

*Calculated using country level values above

^Mean (95% Confidence Interval)

†Median (Range)

‡Average of country specific distributions, thus value varies in simulation

^•^Values from DHS surveys include seeking medical care from a medical facility (including public and private facilities), however does not include care seeking at a pharmacy or traditional healer

**References**

1. Robinson AE. Global trends in care seeking for children with diarrhea: a theoretically grounded exploration of care seeking behavior among the countries of USAID's Demograph Health Surveys. Atlanta, GA: Emory; 2014.

2. Scallan E, Majowicz SE, Hall GV, Banerjee A, Bowman CL, Daly L, et al. Prevalence of diarrhoea in the community in Australia, Canada, Ireland, and the United States. International Journal of Epidemiology. 2005;34:454-60.

3. Chen Y, Yan W-X, Zhou Y-J, Zhen S-Q, Zhang R-H, Chen J, et al. Burden of self-reported actue gastrointestinal illness in China: a population-based survey. BMC Public Health. 2013;13:456.

4. Sang X-L, Liang X-C, Chen Y, Li J-D, Li J-G, Bai L, et al. Estimating the burden of acute gastrointestinal illness in the community in Gansu Province, northwest China, 2012-2013. BMC Public Health. 2014;14:787.

5. Van Cauteren D, De Valk H, Vaux S, Le Strat Y, Vaillant V. Burden of acute gastroenteritis and healthcare-seeking behaviour in France: a population-based study. Epidemiol Infect. 2012;140:697-705.

6. Saha D, Akinsola A, Sharples K, Adeyemi MO, Antonio M, Imran S, et al. Health care utilization and attitudes survey: understanding diarrheal disease in rural Gambia. American Journal of Tropical Medicine and Hygiene. 2013;89(Suppl 1):13-20.

7. Sur D, Manna B, Deb AK, Deen JL, Danovaro-Holliday MC, von Seidlein L, et al. Factors associated with reported diarrhoea episodes and treatment-seeking in an urban slum of Kolkata, India. Journal of Health Population and Nutrition. 2004;22(2):130-8.

8. Simanjuntak CH, Punjabi NH, Wangsasaputra F, Nurdin D, Pulungsih SP, Rofiq A, et al. Diarrhoea episodes and treatment-seeking behaviour in a slum area of North Jakarta, Indonesia. Journal of Health Population and Nutrition. 2004;22(2):119-29.

9. Motlagh ME, Heidarzadeh A, Hashemian H, Dosstdar M. Patterns of care seeking during epidoses of childhood diarrhea and its relation to preventative care patterns: national integrated monitoring and evaluation survey (IMES) of family health Islamic Republic of Iran. International Journal of Preventive Medicine. 2012;3:60-7.

10. Scavia G, Baldinelli F, Busani L, Caprioli A. The burden of self-reported actue gastrointestinal illness in Italy: a retrospective survey, 2008-2009. Epidemiol Infect. 2012;140:1193-206.

11. Tee GH, Kaur G, Ramanathan P, Amal NM, Chinna K. Health seeking behavior among Malaysians with acute diarrheal disease. Southeast Asia Journal of Tropical Medicine and Public Health. 2011;42(2):424-35.

12. Adlam SB, Perera S, Lake RJ, Campbell DM, Williman JA, Baker MG. Acute gastrointestinal illness in New Zealand: a community study. Epidemiol Infect. 2011;139:302-8.

13. Baumann-Popczyk A, Sadkowska-Todys M, Rogalska J, Stefanoff P. Incidence of self-reported actue gastrointestinal infections in the community in Poland: a population-based study. Epidemiol Infect. 2012;140:1173-84.

14. Howidi M, Al Kaabi N, El Khoury AC, Brandtmuller A, Nagy L, Richer E, et al. Burden of acute gastroenteritis among children younger than 5 years of age - a survey among parents in the United Arab Emirates. BMC Pediatr. 2012;12:74.

15. Hall AJ, Rosenthal M, Gregoricus N, Greene SA, Ferguson J, Henao OL, et al. Incidence of acute gastroenteritis and role of norovirus, Georgia, USA, 2004-2005. Emerging Infectious Diseases. 2011;17(8):1381-8.
